# Alkali-Activated Mortars with Recycled Tyre Rubber Aggregates: A Preliminary Mechanical Study

**DOI:** 10.3390/ma19173621

**Published:** 2026-08-26

**Authors:** Ivo Costa, Renato Neves, António Duarte, Miguel Bravo

**Affiliations:** 1 Department of Civil Engineering, Escola Superior de Tecnologia do Barreiro, Universidade Politécnica de Setúbal, 2839-001 Lavradio, Portugal; ivo.costa@estudantes.ips.pt; 2 CERIS—Civil Engineering Research and Innovation for Sustainability, Department of Civil Engineering, Architecture and Environment, Instituto Superior Técnico (IST), Universidade de Lisboa, 1049-001 Lisbon, Portugal; renato.neves@tecnico.ulisboa.pt (R.N.); antonio.duarte@tecnico.ulisboa.pt (A.D.)

**Keywords:** cementitious mortar, fly ash mortar, alkaline activation, recycled tyre rubber, NaOH treatment, sustainability

## Abstract

**Highlights:**

Fly ash alkali-activated mortars (AAM) and cementitious mortars (CM) with recycled tyre rubber (RTR) were tested. Rubber replaced sand at 5–20% (volume) with/without NaOH pre-treatment (saturated solution for 30 min). AAM showed higher strength and lower water absorption than CM under the adopted mix-design and curing conditions.

20% RTR caused major strength/stiffness losses, whereas 5–10% RTR was more viable.

NaOH rubber treatment provided no consistent benefit and overall reduced mechanical performance.

**What are the main findings?**

- Reference AAM reached 46–53 MPa, whereas reference CM reached 28–31 MPa (28–56 days).
- RTR (5–20%) reduced strength/stiffness/UPV, while losses were smaller in AAM.
- NaOH-treated rubber mortars showed no benefit and often decreased strengths (except at 5% RTR).

**What are the implications of the main findings?**

- Under the adopted mix-design and curing conditions, AAM showed higher overall mechanical performance than CM for the tested RTR contents.
- The use of moderate RTR contents (5–10%) provides a balance of weight reduction and mechanical needs.
- RTR treatment needs to be optimised with alternative chemical/mechanical processes.

**Abstract:**

This preliminary experimental study investigates mortars with partial replacement of natural sand by recycled tyre rubber (RTR) and alkali-activated fly ash as an alternative binder to Portland cement. An experimental programme was carried out on 14 mortar mixes, including cementitious mortars (CM) and alkali-activated mortars (AAM), with 5%, 10% and 20% RTR incorporation and with/without NaOH pre-treatment of rubber (saturated solution for 30 min). Fresh behaviour was assessed through flow and fresh density, while mechanical-related performance was evaluated at 28 and 56 days through compressive and flexural strength, modulus of elasticity and ultrasonic pulse velocity. Physical properties included open porosity, dry density, water absorption by immersion and capillarity. The alkali-activated reference mix achieved higher compressive strengths (46.1–53.0 MPa) than the cement reference (28.4–30.6 MPa) under the adopted mix-design and curing conditions. RTR incorporation reduced stiffness and strength in all specimens, with 20% RTR decreasing flexural strength by 26.6–51.4%, although losses were generally smaller in AAM than in CM. Under the treatment condition investigated, NaOH pre-treatment did not consistently improve mechanical-related performance, except for limited gains at 5% RTR. Overall, AAM with 5–10% RTR showed a better balance between density reduction and mechanical-related performance than CM.

## 1. Introduction

Lowering the environmental impact of the construction sector has become one of the main priorities in Civil Engineering research. Ordinary Portland cement (OPC) remains a major contributor to the embodied carbon of construction materials, with about 750 kg of CO_2_ emissions associated with the production of each tonne of cement [1]. In response, decarbonisation strategies increasingly focus on reducing OPC consumption through alternative binders and the incorporation of industrial by-products and recycled materials into cementitious and non-cementitious composites [2]. Among these approaches, alkali-activated materials can be produced from aluminosilicate precursors derived from industrial processes, such as fly ash, activated using alkaline solutions and thereby reducing reliance on clinker-based binders [3].

In parallel with the cement-related challenge, the management of end-of-life tyres remains an important environmental issue. Around one billion tyres are estimated to reach the end of their service life globally each year, with a significant proportion still being landfilled without treatment [4]. This has encouraged research into recycling routes such as the use of recycled tyre rubber (RTR) as a partial replacement of natural aggregates in concrete and mortars. RTR incorporation can reduce the demand for natural resources and generally decreases material density, while increasing deformability and energy-absorption capacity. However, numerous studies report reductions in mechanical performance as rubber content increases, commonly associated with the lower stiffness of rubber and weak bonding between rubber particles and the surrounding matrix [5].

Within this context, the present study considers the partial replacement of natural aggregates by RTR, with and without NaOH pre-treatment, in both fly ash alkali-activated mortars and cementitious mortars. This combination raises questions about how rubber performs in alkali-activated systems containing NaOH and Na_2_SiO_3_ and whether surface-treatment strategies commonly adopted in cement-based systems can also be applied to these binders.

A broad consensus in the literature indicates that replacing natural aggregates with rubber tends to reduce compressive strength, tensile or flexural strength, and modulus of elasticity. Elchalakani [6] associates this behaviour mainly with two mechanisms: the large stiffness difference between rubber particles and the surrounding hardened matrix and the weak interfacial transition zone (ITZ). Under load, rubber particles deform more than mineral aggregates, which can generate local stress concentrations in the surrounding paste. In addition, the hydrophobic surface of rubber can limit wetting and reduce adhesion at the interface [6]. Similar observations have been reported by Uygunoğlu and Topçu [7] and Pedro et al. [8], who described weaker or more discontinuous regions around rubber particles in rubberised cementitious materials. The poor interaction between rubber and cementitious matrices has also been linked to zinc stearate present at the tyre-rubber surface, which may contribute to its water-repellent character [5,9].

To mitigate these effects, several studies have investigated chemical or physical surface treatments intended to improve rubber-matrix adhesion. NaOH treatment is among the most frequently studied approaches and has been reported to increase rubber-surface hydrophilicity and modify its surface characteristics [10]. Guo et al. [11] related ITZ behaviour to wetting and water-film effects at the aggregate surface and suggested that changes in surface chemistry and roughness may influence adhesion. Nevertheless, the effectiveness of NaOH treatment depends on several variables, including concentration, treatment duration, rubber characteristics, and binder type. Youssf et al. [9], using a 10% NaOH solution and 20% sand replacement by rubber, compared treatment durations of 0.5, 1, and 2 h. At 28 days, compressive-strength increases of 17.3%, 7.5%, and 3.6%, respectively, were reported relative to untreated rubber, leading the authors to identify 0.5 h as the most effective treatment duration under their experimental conditions. These results show that longer or harsher treatment does not necessarily lead to greater improvement.

RTR incorporation also affects fresh-state properties. Mohammadi et al. [5] reported an increase in the water-reducer dosage required to maintain a target slump of 60 ± 10 mm as rubber content increased. For mixtures with w/c = 0.40, the dosage increased from 3.20 L/m^3^ in the reference concrete to 4.44 L/m^3^ at 40% crumb rubber, while for w/c = 0.45 it increased from 1.15 to 2.07 L/m^3^. In fly ash geopolymer mortars, Abd-Elaty et al. [12] reported flow reductions ranging from approximately 5% to 55%, with the greatest reduction occurring for the finest rubber fraction at 30% sand replacement. These results indicate that workability changes depend strongly on rubber content, particle size, mixture composition, and admixture dosage.

A consistent effect of rubber incorporation is the reduction in fresh and hardened density because rubber is considerably lighter than natural mineral aggregates. Pedro et al. [8] reported bulk densities of 451 kg/m^3^ for rubber and 1451 kg/m^3^ for river sand. In the same study, fresh mortar density decreased by approximately 4%, 7%, and 10% for 5%, 10%, and 15% rubber replacement, respectively, while 28-day dry density decreased by 4.32%, 7.45%, and 9.90%. Bravo and de Brito [13] similarly observed fresh-density reductions of approximately 7–8% in concrete at 15% tyre-aggregate replacement, depending on the aggregate fraction replaced. Thus, the decrease in density is one of the most consistent physical effects associated with RTR incorporation.

Mechanical-property reductions generally become more pronounced as rubber content increases. Pedro et al. [8] reported 28-day flexural-strength reductions of approximately 12%, 5%, and 21% for mortars containing 5%, 10%, and 15% rubber, respectively; at 90 days, the corresponding reductions were 34%, 19%, and 38%. The compressive-strength reduction reached about 55% at 28 days and 60% at 90 days for 15% rubber replacement. Mohammadi et al. [5] found that each 10% increase in rubber content produced an approximate 17% reduction in compressive strength and an 8% reduction in flexural strength. After optimisation of the NaOH treatment, however, treated-rubber concrete achieved 25% higher compressive strength and 5% higher flexural strength than the corresponding untreated-rubber concrete. These results indicate that treatment may improve mechanical performance under specific conditions, but its effectiveness cannot be assumed independently of the adopted treatment procedure.

A similar trend is observed for stiffness. Pedro et al. [8] reported reductions in modulus of approximately 12%, 28%, and 32% at 28 days for 5%, 10%, and 15% rubber replacement, respectively, increasing to about 20%, 33%, and 43% at 90 days. Uygunoğlu and Topçu [7] also reported substantial modulus reductions for mortars with 50% rubber replacement, ranging from 47.4% to 68.4% for the different w/c ratios considered. This behaviour is consistent with the much lower stiffness of rubber relative to mineral aggregates and with the greater heterogeneity of rubberised mixtures, and may also be reflected in ultrasonic pulse velocity (UPV).

Rubber incorporation can also modify porosity and water transport. Martins [14] noted the correlation between cement hydration, pore filling and porosity development over time. In rubberised self-consolidating mortars, Uygunoğlu and Topçu [7] reported that, for mixtures with a water-to-powder ratio (w/p) of 0.47, apparent porosity increased from approximately 23% in the reference mix to about 29–30% for 10–20% rubber replacement, reaching around 41% at the highest replacement levels. Bravo and de Brito [13] also observed an increase of about 15% in water absorption by immersion at 15% tyre-aggregate replacement. These variations were associated with changes in water demand, compaction and void content.

Capillary absorption may follow a different trend because of the hydrophobic nature of rubber. Segre and Joekes [10] observed lower water uptake in cement pastes containing 10% tyre rubber by mass than in the reference paste, with NaOH-treated rubber showing the lowest absorption. Souza [15] reported that 48 h capillary absorption decreased from 1.335 g/cm^2^ in the reference mortar to 0.132, 0.122, and 0.349 g/cm^2^ for 10%, 20%, and 30% rubber incorporation, corresponding to reductions of approximately 90%, 91%, and 74%. In the same study, UPV decreased from 3305 m/s in the reference mortar to 2738, 2104, and 1399 m/s as rubber content increased. These results illustrate that reduced capillary uptake can coexist with lower wave-transmission velocity in rubberised mortars.

Alkali-activated systems can exhibit different mechanical-development kinetics from OPC-based materials. Carvalho et al. [16] reported that, between 7 and 28 days, flexural strength increased from 5.35 to 6.89 MPa in cement mortars (+28.8%) and from 4.19 to 8.52 MPa in fly ash alkali-activated mortars (+103.3%). Modulus increased from 34.93 to 37.37 GPa (+7.0%) and from 16.86 to 22.82 GPa (+35.3%), respectively. Hardened density showed only limited variation over the same period. Suescum-Morales et al. [3] also observed increasing UPV with age in alkali-activated fly ash mortars; for the reference mix, UPV increased from 3509 to 3615 m/s between 7 and 28 days, while increases of about 6.0% and 4.9% were reported for mixtures containing 10% and 20% MgO, respectively. These studies indicate that the evolution of mechanical-related and physical properties in alkali-activated mortars may differ from that of conventional cement mortars.

Studies specifically combining rubber with alkali-activated or geopolymer binders remain less common. Abd-Elaty et al. [12] reported decreasing mechanical strength with increasing rubber content in geopolymer composites, but also improved post-cracking behaviour. The toughness index increased by approximately 12%, 18%, and 26% with 3%, 6%, and 9% crumb rubber, respectively, while the use of 20% crumb rubber with 1% polypropylene fibres increased flexural toughness by 27.5% compared with the fibre-only mixture. These results indicate that rubber incorporation may involve a trade-off between peak mechanical properties and deformation or energy-absorption capacity.

Although several studies have addressed RTR incorporation in cement-based materials and others have investigated fly ash alkali-activated mortars, the combined use of RTR, including NaOH-treated rubber, in alkali-activated mortars remains less explored. Accordingly, the present work is intended as a preliminary experimental study focused primarily on the mechanical-related performance of alkali-activated and cementitious mortars incorporating 5%, 10%, and 20% RTR as partial replacement of natural sand, with and without NaOH pre-treatment. Mechanical-related performance was assessed through compressive and flexural strength, dynamic modulus of elasticity and ultrasonic pulse velocity, complemented by fresh-state and basic physical tests. No microstructural, mineralogical, or chemical characterisation, such as SEM or XRD, was performed. The study is thus intended to establish preliminary performance trends and identify aspects requiring further investigation.

## 2. Materials and Methods

### 2.1. Materials

#### 2.1.1. Precursors

The precursor used in cement-based mixes was Portland cement CEM II/B-L 32.5 N produced by the company ‘Secil’ (Lisbon, Portugal), following the NP EN 197-1 [17] standard, with 21–35% by mass of limestone, with a compressive strength at 28 days of 32.5 MPa and a bulk density of 3100 kg/m^3^.

The precursor used in alkali-activated mixes was class F coal fly ash (FA) supplied by the company ‘EDP—Gestão da Produção de Energia, S.A.’ (Lisbon, Portugal) at the Sines thermoelectric power plant, with a bulk density of 2431 kg/m^3^ and a total CaO content below 10%.

#### 2.1.2. Aggregates

The natural aggregates used were fine sand (0 to 2 mm) and coarse sand (0 to 4 mm) and were obtained through the company ‘Grupo SOARVAMIL’ (Corroios, Portugal). An oven-dried density of 2630 kg/m^3^, water absorption of 0.4% and 0.5%, respectively, and water absorption at 10 min of 50% and 60%, respectively, were considered.

The recycled tyre rubber aggregates were obtained through a mechanical process of crushing used tyres. These recycled aggregates, with an average bulk density of 1100 kg/m^3^, were subjected to a process to optimise their granulometric curve, separating them by sieves into three particle-size fractions (0–1 mm, 1–2 mm, and 2–4 mm) to replace 5%, 10%, and 20% of the natural aggregates by volume.

#### 2.1.3. Alkaline Activator Solution

Sodium hydroxide (NaOH) and sodium silicate (Na_2_SiO_3_) were used as components for the alkaline activator solution. The NaOH supplied contained 99% pure NaOH pellets and a density of 2130 kg/m^3^, also used for the RTR treatment. The Na_2_SiO_3_ was supplied in solution with a density of 1370 kg/m^3^, containing 28% SiO_2_, 8% Na_2_O, and 64% H_2_O, by mass. A water-reducing superplasticiser (SikaPlast-717) with a pH of 10 was used as an admixture in the mortar design at a concentration of 0.2% by precursor mass and was supplied by the company ‘Sika’ (Vila Nova de Gaia, Portugal).

#### 2.1.4. Recycled Tyre Rubber Treatment with NaOH

In this study, mortars were carried out with volume replacements of 5%, 10%, and 20% using rubber pre-treated under one condition: immersion in a saturated NaOH solution for 30 min. The resulting mortars were compared with equivalent mixes containing untreated rubber. The 30 min duration is consistent with the 0.5 h treatment period investigated by Youssf et al. [9], although the NaOH concentration and other treatment parameters differ; therefore, the present conclusions apply only to the treatment condition tested here.

**Figure 1:**
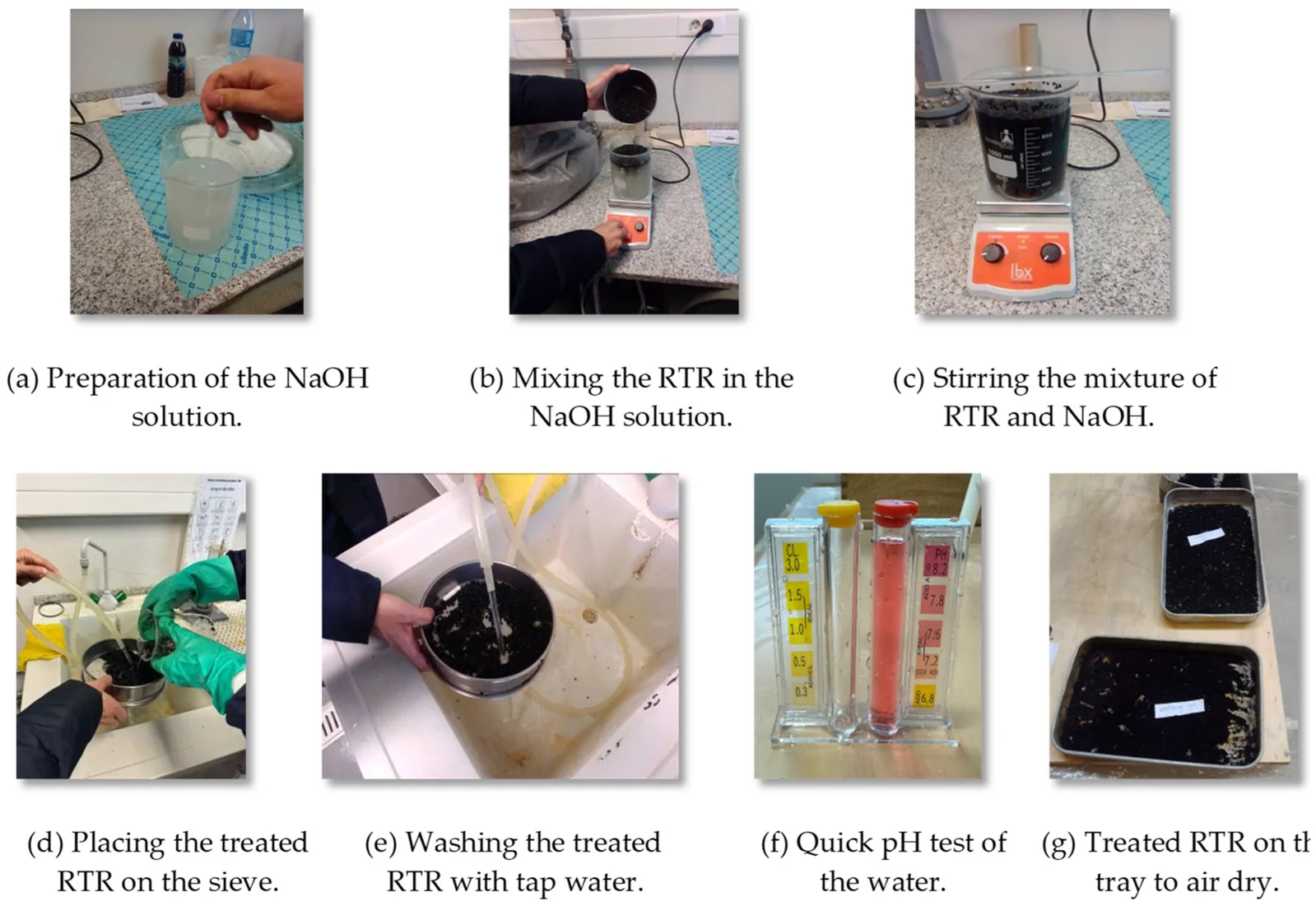
Procedure for recycled tyre rubber treatment in NaOH solution.

The procedure adopted for treating the rubber with NaOH began by preparing a saturated alkaline solution with water and NaOH in a measuring cup and stirring until the NaOH was completely dissolved (Figure 1a). The solution was then transferred to a magnetic stirrer. The sieved rubber was added to the alkaline solution and stirred until completely submerged (Figure 1b). The mixture was kept under stirring for 30 min (Figure 1c). It was then poured onto a 75-micrometre mesh sieve, which retained the rubber particles while the alkaline solution drained through the mesh (Figure 1d). The retained rubber was washed with pH-neutral water (Figure 1e) until the pH of the residual wash water indicated that residual NaOH had been removed (Figure 1f). Finally, the treated rubber was removed from the sieve, spread on a tray, air-dried at room temperature for 5 days, and then incorporated into the mortars (Figure 1g). The process is illustrated in Figure 1.

### 2.2. Mix Design and Curing Conditions

The experimental campaign was organised in two phases. The first phase of the laboratory work began with the characterisation (bulk density, particle size distribution, water absorption, and density) of the materials (cement, fly ash, aggregates, and rubber aggregates) and the production of preliminary mixtures of reference mortars (cement and fly ash) without recycled rubber aggregates, in order to determine the w/b ratios that lead to a flow of 150 ± 10 mm.

In a second phase, six prismatic specimens (40 mm × 40 mm × 160 mm) were produced from each mortar mixture (already taking into account the appropriate w/b ratios): cement mortars or alkali-activated mortars with sand replaced by rubber aggregates (0%, 5%, 10%, and 20%) with or without NaOH treatment, representing a total of 84 specimens, as shown in Table 1.

The composition of the reference cement mortar (RCM) required a w/b ratio of 0.53, while the reference alkali-activated mortar used a w/b ratio of 0.38, both selected to obtain a flow of 150 ± 10 mm. A constant precursor content of 430 kg/m^3^ (cement or fly ash) was used. For AAM, the alkaline solution used a Na_2_O concentration of 13% and a SiO_2_/Na_2_O mass ratio of 1.0. The water-reducing admixture dosage was 0.2% by precursor mass. RTR replaced the corresponding sand fraction by volume, using the three particle-size fractions described in Section 2.1.2. Because w/b ratio and curing regime were not held constant between CM and AAM, comparisons between the two binder families represent the performance of the adopted systems and should not be interpreted as isolating binder chemistry alone. Table 2 and Table 3 present the design composition of all mortar mixes, respectively, for cementitious and alkali-activated mortar mixes.

The mortar production process comprised the following sequence: (i) all constituent materials were weighed using a digital balance with a precision of 0.1 g; each batch corresponded to 2 L of mortar, sufficient to produce six specimens (note that, for each testing age, three specimens were allocated to each test and that each specimen was used for only one test, except for non-destructive tests, namely the dynamic modulus of elasticity and UPV); (ii) for the alkali-activated mortars, the activating solution was prepared by first combining water with Na_2_SiO_3_, followed by the addition of NaOH, with the solution manually stirred using a glass rod for 5 min until dissolution, after which the superplasticiser was added; for the cement mortars, only water and superplasticiser were premixed and manually stirred; (iii) the mixer bowl was slightly moistened and the precursor, either cement or fly ash, was introduced; (iv) water, in the case of cement mortars, or the alkaline activating solution, in the case of alkali-activated mortars, was then added; (v) the bowl was fitted to the rotary mixer and the automatic mixing programme was started; (vi) the mixture was mixed at low speed for 30 s, after which the fine aggregates were added and mixing continued at low speed for a further 30 s; (vii) after a total mixing time of 60 s, the mixer was switched to high speed for 30 s; (viii) mixing was then stopped to manually scrape and reincorporate any material adhering to the walls and bottom of the bowl; and (ix) after a 60 s resting period, mixing was resumed at high speed for a final 60 s.

After production, all CM mixes were kept under laboratory curing conditions (20 ± 5 °C and 60 ± 10% relative humidity) until testing at 28 and 56 days, with specimens demoulded after 24 h. AAM mixes were thermally cured at 70 °C for 24 h and then stored under laboratory conditions until testing. These different curing regimes form part of the adopted mix systems and are a limitation when directly comparing CM and AAM.

### 2.3. Test Methods

All tests carried out in the experimental campaign are listed in Table 4, along with the corresponding standards followed. This combination of tests enabled a comprehensive characterisation of the components, such as the precursors and aggregates, as well as the assessment of the fresh-state performance, the mechanical strength and the physical properties of the mortar mixes. However, comparisons in Section 3 are descriptive, as no inferential statistical significance is claimed.

## 3. Results and Discussion

The results of the experimental campaign are presented in this section, covering the physical characterisation of the raw materials, as well as the fresh-state, mechanical-related and physical properties of the mortars. The results are then discussed and compared with relevant findings from the literature.

### 3.1. Physical Properties of Raw Materials

In the first stage of this study, the particle size distribution, densities, and water absorption of the aggregates were assessed to examine their suitability and potential influence when natural sand was partially replaced by RTR. The results of the physical characterisation of the raw materials are presented in this section.

#### 3.1.1. Particle Size Distribution of Aggregates

**Figure 2:**
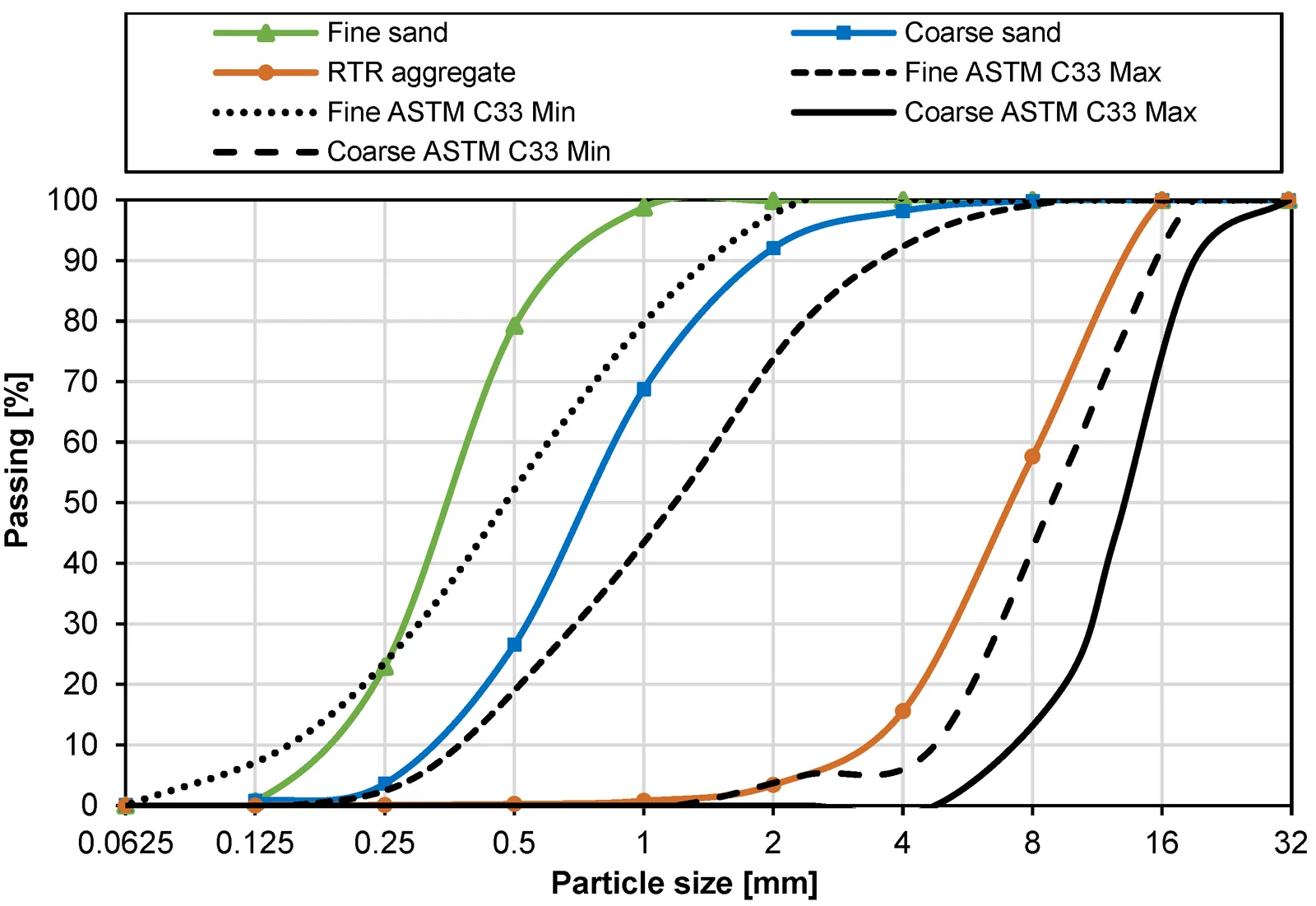
Aggregate particle size distribution curves.

The results obtained in the granulometric analysis of natural sand and RTR are shown in Figure 2. It should be noted that the rubber sample used is in the same condition as after mechanical grinding, and therefore still contains particles larger than 4 mm.

Figure 2 shows that the as-received rubber had a particle size distribution different from those of the natural sands and closer to coarse aggregate according to the ASTM C33 reference curves. For this reason, the rubber used for mortar production was sieved and divided into three fractions (0–1 mm, 1–2 mm, and 2–4 mm) before replacing 5%, 10%, and 20% of the sand volume. This procedure produced an RTR grading closer to that of the natural sand used in the mixtures.

#### 3.1.2. Bulk Density of Aggregates and Precursors

For sand and rubber aggregates, as well as for cement and fly ash precursors, bulk density was determined experimentally. The final bulk density corresponds to the average of three readings, according to the test results presented in Table 5.

It was found that the bulk density of rubber, as expected, is lower than that of sand. Thus, partially replacing the sand aggregate with rubber results in lighter mortars.

#### 3.1.3. Density and Water Absorption of Aggregates

For this test, three types of density were determined for the aggregates: the apparent density (ρ_a_), the real or oven-dry density (ρ_rd_), and the saturated surface density (ρ_ssd_). Table 6 shows the results for aggregate density and water absorption (WA), highlighting the higher densities of the sands relative to rubber.

The test yielded an apparent 24 h water absorption of 3.7% for RTR, higher than that measured for the sands. This result should be interpreted cautiously because tyre rubber is generally described as hydrophobic [9,11], and the standard aggregate procedure may include water retained on the irregular rubber surface when the saturated-surface-dry mass is determined. No independent surface-wetting or adsorption test was performed in this study; therefore, the measured value is reported as an apparent water-uptake result rather than evidence of a specific absorption or adsorption mechanism.

### 3.2. Fresh-State Properties of Mortars

The results and analysis of the fresh-state properties for all mortar mixes are described in this section.

#### 3.2.1. Flow

**Figure 3:**
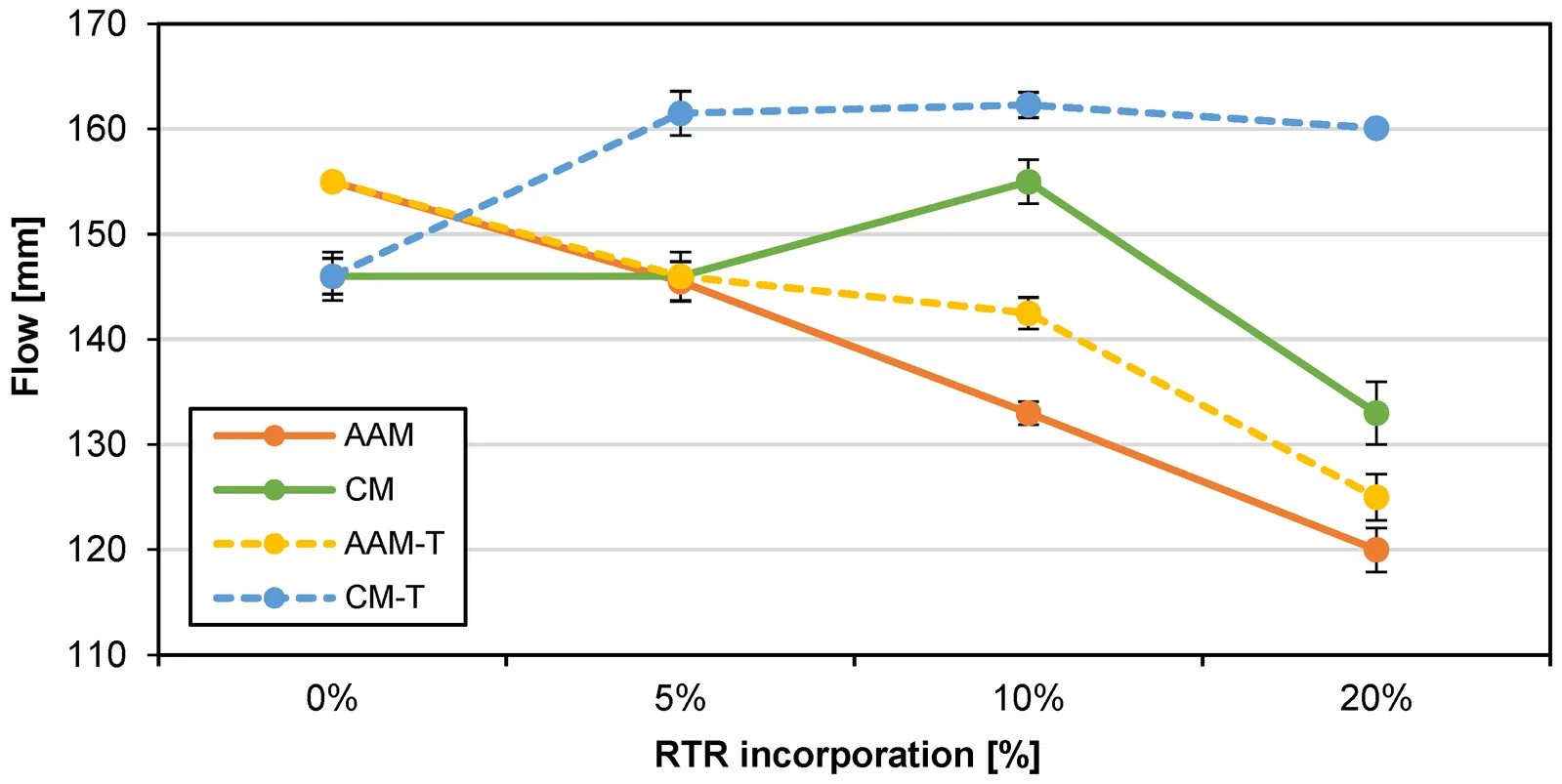
Flow for mortar mixes.

The results of the flow test are shown in Figure 3. Increasing RTR content in AAM reduced flow by 6.1%, 14.2%, and 22.6% for 5%, 10%, and 20% RTR, respectively. This trend is consistent with previous reports linking rubber incorporation to changes in mixture viscosity, particle shape, and packing [5,12]. In CM, the changes were 0.0%, +6.2%, and −8.9% for 5%, 10%, and 20% RTR, respectively, and no monotonic trend was observed.

For NaOH-treated RTR, flow was between 0.4% and 20.4% higher than in the corresponding untreated mixes. Because no statistical significance testing was performed, these differences are interpreted descriptively and do not establish a general effect of NaOH treatment on workability.

#### 3.2.2. Fresh Density

According to the fresh-density results in Table 7, RAAM had a fresh density of 2219.7 kg/m^3^, while RCM had 2188.0 kg/m^3^, a difference of 1.4%. Under the adopted mix designs, all AAM and AAM-T mixes had higher fresh density than the corresponding CM mixes. The different water contents and mix compositions may contribute to this difference; the comparison does not isolate the effect of binder type.

The results also show that density decreases as the RTR content in the mortars increases (average of −2.1%, −4.3%, and −8.8%, respectively, for 5%, 10%, and 20% RTR incorporation), with the decrease relative to the reference mortar more pronounced in cement mortars than in alkali-activated mortars. The density of rubber is lower than the density of sand, so it is logical that this results in a tendency for the density of mortars to decrease [8,13].

The density differences between mortars with treated and untreated rubber ranged from −3.5% to +0.6%. Within the present descriptive dataset, no consistent influence of NaOH treatment on fresh density was identified.

### 3.3. Mechanical-Related Properties of Mortars

The results and analysis of the mechanical-related properties for all mortar mixes are described in this section.

#### 3.3.1. Compressive Strength

**Figure 4:**
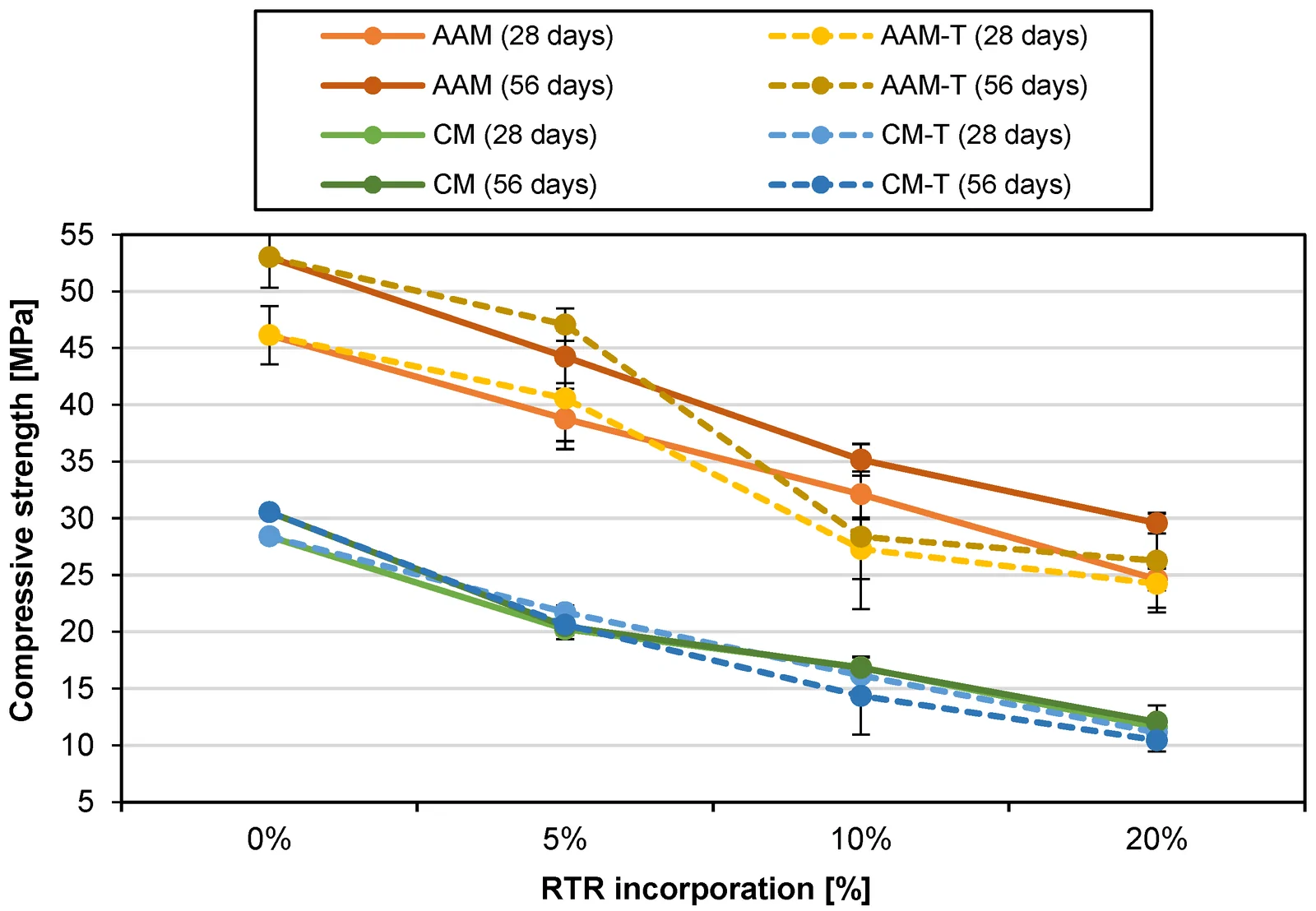
Compressive strength for mortar mixes.

The compressive-strength results are shown in Figure 4. Under the adopted mix-design and curing conditions, RAAM had higher compressive strength (46.14 and 53.01 MPa at 28 and 56 days) than RCM (28.41 and 30.55 MPa). The different w/b ratios and curing regimes may contribute to this difference, so it cannot be attributed to binder chemistry alone. Compressive strength decreased with RTR incorporation; at 20% RTR, values were 44.2–60.8% below the corresponding reference mortar. This trend is consistent with mechanisms reported in the literature, including the stiffness contrast between rubber and mineral aggregate and weaker rubber-matrix interfacial bonding [8,12], but the ITZ was not directly characterised in this study. The reductions in AAM and AAM-T (average −13.9%, −37.9%, and −47.2% for 5%, 10%, and 20% RTR) were smaller than those in CM and CM-T (average −29.4%, −45.4%, and −61.6%). Thus, within the tested systems, AAM retained a larger proportion of its reference compressive strength after RTR incorporation.

From 28 to 56 days, compressive strength changed by an average of +3.1% in CM and −3.7% in CM-T, whereas it increased by +14.6% in AAM and +10.8% in AAM-T. This later strength development is consistent with trends reported for alkali-activated fly-ash mortars [16]. No XRD or other phase-characterisation test was performed, so the evolution cannot be directly assigned to specific reaction products in the present study.

For NaOH-treated RTR, except at 5% RTR, compressive strength generally decreased relative to the corresponding untreated mixes at 28 and 56 days (average changes of −4.1%, −14.0%, −8.3%, and −15.3% for CM-T (28 d), CM-T (56 d), AAM-T (28 d), and AAM-T (56 d), respectively). This differs from studies in which optimised NaOH treatment improved strength. For example, Mohammadi et al. [5] reported approximately 25% higher compressive strength after optimising the treatment, while Youssf et al. [9] reported the greatest benefit for a 0.5 h treatment under their conditions. Mohammadi et al. [5] also discussed surface porosity and air entrapment as possible causes of strength loss after non-optimised treatment. Since SEM, surface roughness and pore-structure measurements were not performed here, such mechanisms remain hypotheses rather than direct observations. At 5% RTR, the average changes were +7.4%, +0.7%, +4.7%, and +6.4% for the same four comparisons.

#### 3.3.2. Flexural Strength

**Figure 5:**
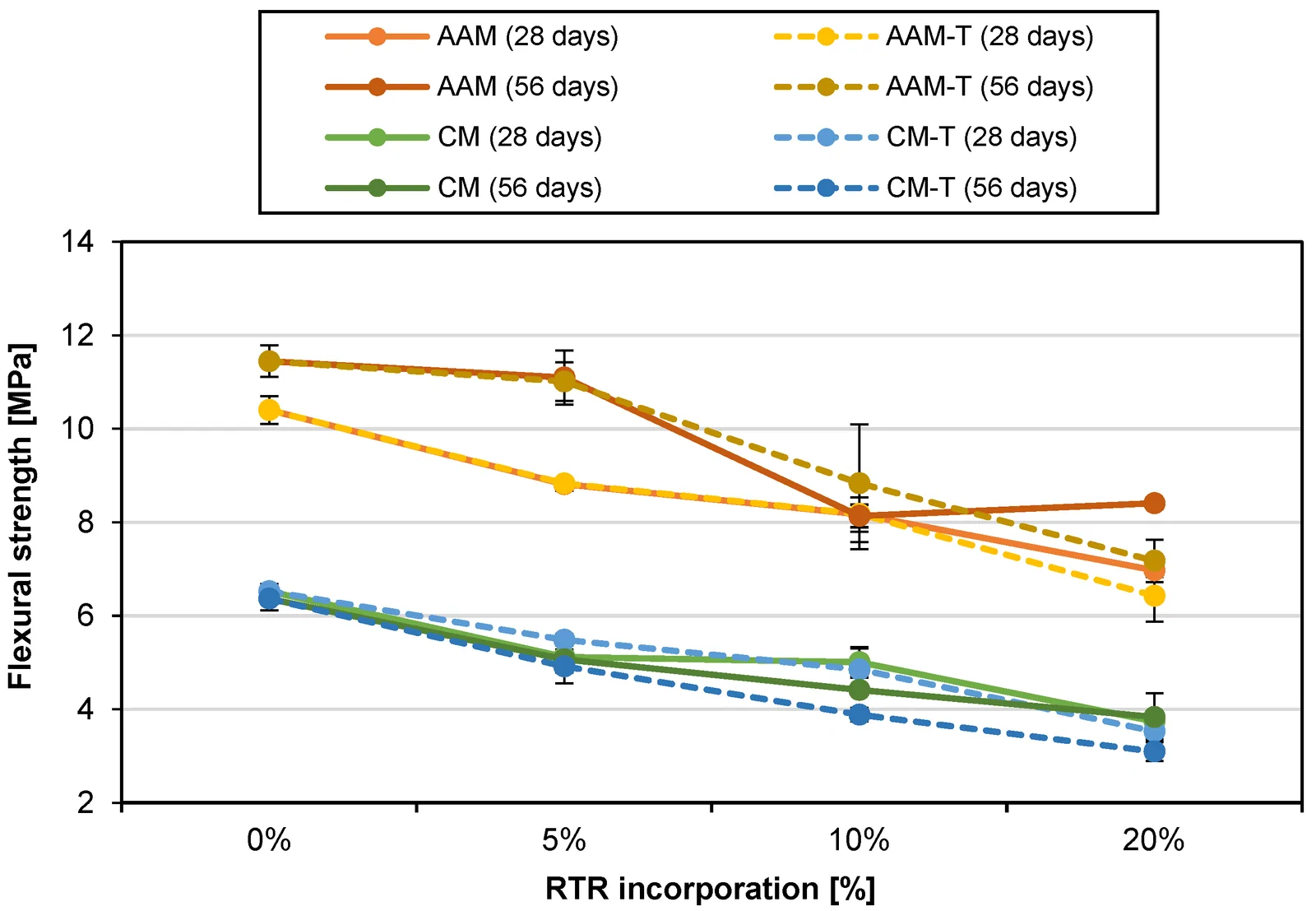
Flexural strength for mortar mixes.

The flexural-strength results are presented in Figure 5. Under the adopted experimental conditions, RAAM had higher flexural strength (10.40 and 11.45 MPa at 28 and 56 days) than RCM (6.52 and 6.37 MPa). As explained for compressive strength, this comparison is also affected, for flexural strength, by differences in w/b ratio and curing regime. Flexural strength decreased with RTR incorporation, in agreement with Pedro et al. [8] and Abd-Elaty et al. [12]; at 20% RTR, values were 26.6–51.4% below the corresponding reference mortar. Previous studies relate this trend to rubber-matrix interfacial bonding and the stiffness contrast between rubber and mineral aggregates. These mechanisms were not directly characterised here and are used only to interpret the measured trend.

It was also possible to verify that AAM and AAM-T always present lower reduction values (average of −9.3%, −23.6%, and −33.8%, respectively, for 5%, 10%, and 20% RTR incorporation), compared to CM and CM-T (average of −20.2%, −29.6%, and −44.9%, respectively, for 5%, 10%, and 20% RTR incorporation).

From 28 to 56 days, flexural strength decreased in CM and CM-T by an average of 3.2% and 11.3%, respectively. Shrinkage-related microcracking is one possible explanation reported for such behaviour, but shrinkage and microstructure were not measured in this study, and this mechanism cannot be confirmed. In AAM and AAM-T, flexural strength increased by an average of 14.0% and 13.6%, respectively, consistent with later mechanical development in these alkali-activated mixes.

When treated and untreated RTR mixes were compared, flexural strength changed by an average of −0.5%, −11.4%, −2.5%, and −2.3% for CM-T (28 d), CM-T (56 d), AAM-T (28 d), and AAM-T (56 d), respectively. These results do not reproduce the improvements reported for some optimised NaOH treatments [5,9]. Surface porosity and air entrapment have been proposed in the literature as possible explanations for strength losses after treatment [5], but no SEM, surface-characterisation or pore-structure measurements were performed here. Therefore, this explanation remains hypothetical.

#### 3.3.3. Modulus of Elasticity

**Figure 6:**
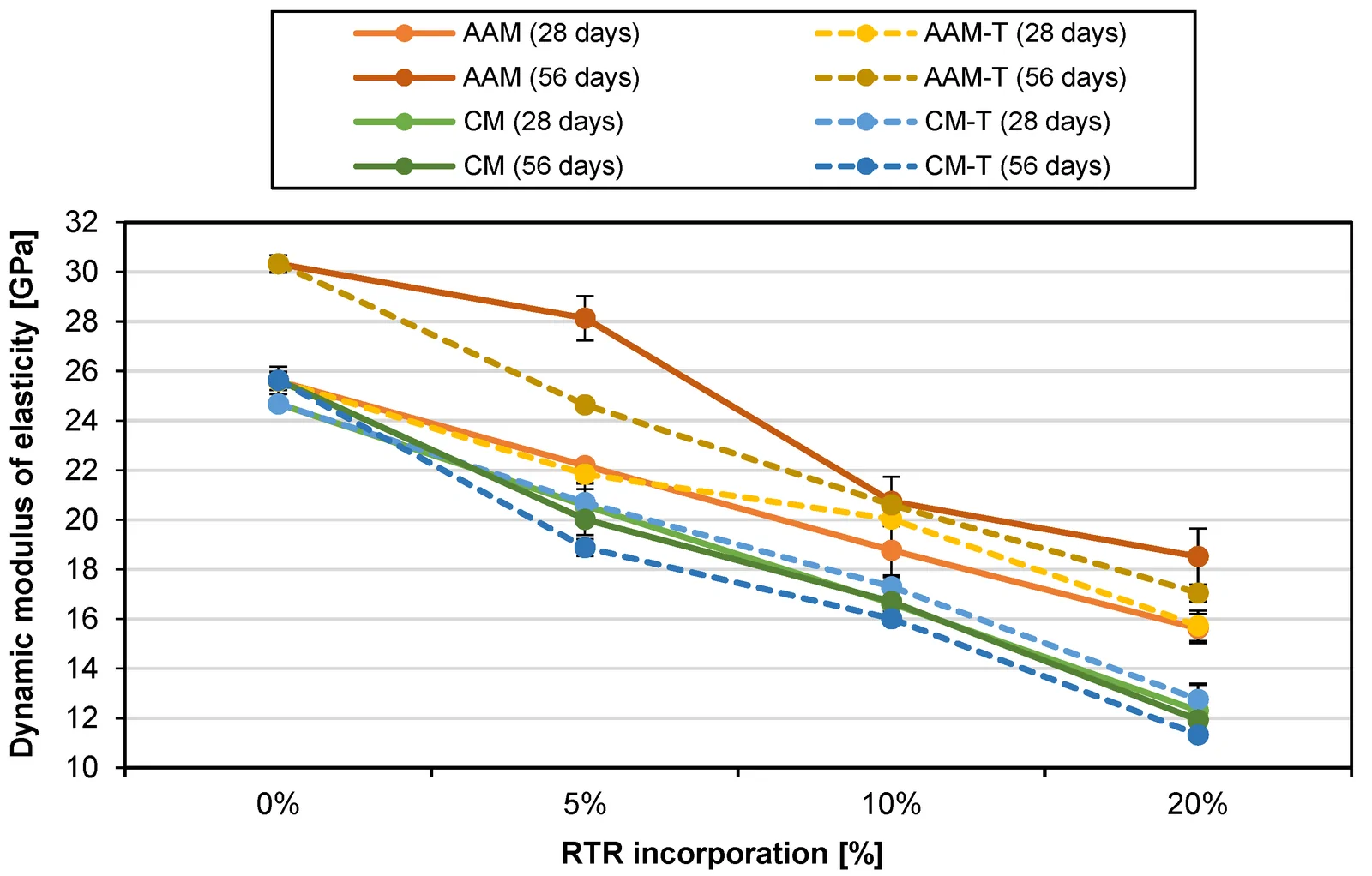
Modulus of elasticity for mortar mixes.

The dynamic modulus of elasticity (DME) results are summarised in Figure 6. RAAM had DME values 3.7% and 18.3% higher than RCM at 28 and 56 days, respectively, under the adopted mix-design and curing conditions. DME decreased with increasing rubber content; at 20% RTR, values were 38.6–55.8% below the corresponding reference mortar. This trend is consistent with the lower stiffness of rubber aggregates and with porosity/air-content effects reported by Pedro et al. [8]. AAM and AAM-T retained higher DME values than CM and CM-T in the present experimental systems, but the comparison is influenced by their different w/b ratios and curing regimes.

From 28 to 56 days, DME changed by an average of −0.4% in CM and −5.9% in CM-T, whereas it increased by +18.6% in AAM and +10.6% in AAM-T. The latter trend is consistent with later mechanical development reported for alkali-activated systems [16], without implying direct identification of the reaction products responsible.

At 28 days, DME of treated-rubber mixes was on average 2.8% higher in CM-T and 2.0% higher in AAM-T than in their untreated counterparts. At 56 days, the corresponding changes were −5.0% and −7.0%. These variations are limited and do not show a consistent treatment effect across age and binder family; therefore, the influence of NaOH treatment on DME remains inconclusive.

**Figure 7:**
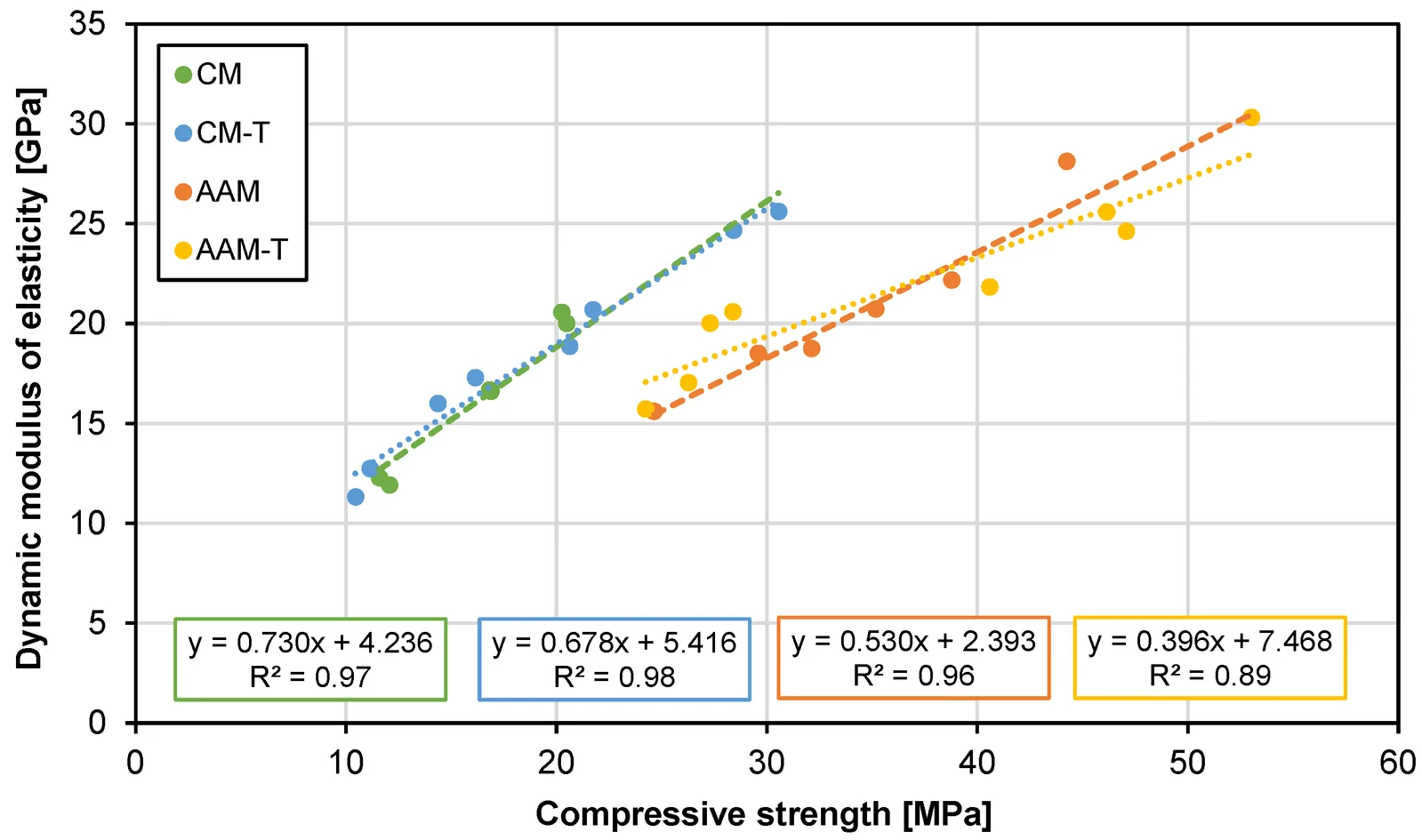
Correlation between compressive strength and modulus of elasticity results.

Linear regressions were obtained for CM, CM-T, AAM, and AAM-T to describe the correlation between compressive strength and modulus of elasticity, with R^2^ values ranging from 0.89 to 0.98 (Figure 7). The cementitious mortar groups showed steeper fitted slopes than the alkali-activated groups.

#### 3.3.4. Ultrasonic Pulse Velocity

**Figure 8:**
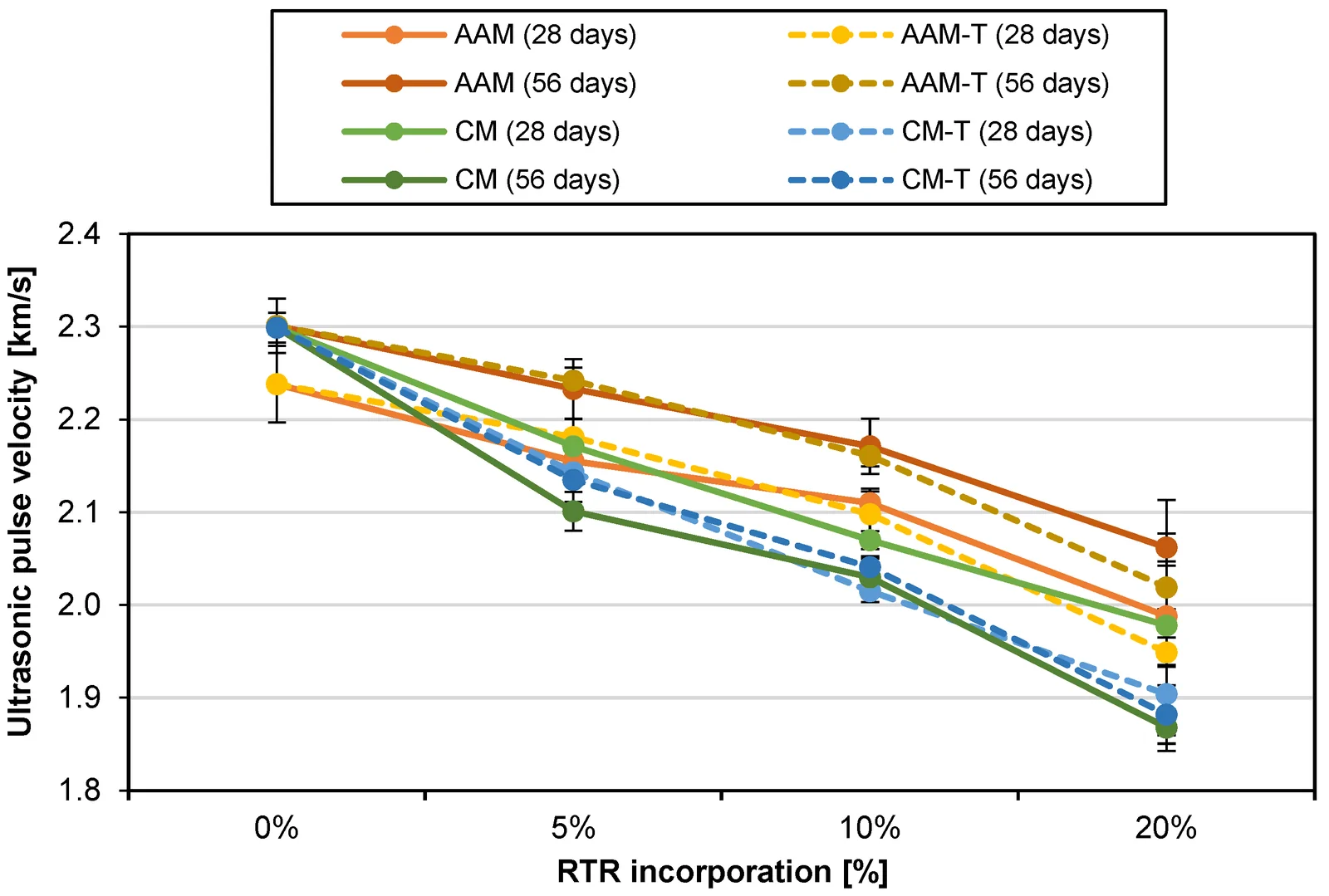
Ultrasonic pulse velocity (UPV) for mortar mixes.

The ultrasonic pulse velocity (UPV) results are summarised in Figure 8. RCM and RAAM values at 28 and 56 days ranged from 1868 to 2301 m/s. These absolute values are relatively low compared with dense structural mortars, and direct comparison between studies should be made cautiously because UPV is influenced by composition, porosity, moisture condition, coupling, and test path. Souza [15], for example, reported values decreasing from 3305 to 1399 m/s as rubber content increased. In the present study, UPV decreased on average by 5.0%, 8.6%, and 14.3% for 5%, 10%, and 20% RTR incorporation, respectively. This trend is consistent with the lower stiffness of rubber and with increased heterogeneity/void content reported for rubberised mortars [7,15]. Because all mixes were tested using the same procedure, the relative trends within this experimental programme are more informative than the absolute classification of the measured velocities.

At 56 days, AAM and AAM-T gave the highest UPV range (2019–2301 m/s), while CM and CM-T ranged from 1868 to 2299 m/s. These results are consistent with differences in the physical structure of the tested mixes, including the lower w/b ratio of AAM, but no microstructural test was performed to demonstrate a denser or more homogeneous matrix directly.

From 28 to 56 days, UPV increased by an average of 3.3% in AAM and 3.1% in AAM-T, while it changed by −2.7% in CM and −0.1% in CM-T. The AAM trend is consistent with the age-related UPV development reported by Suescum-Morales et al. [3] and with the mechanical development discussed by Carvalho et al. [16]. The magnitude of the changes is small, and no direct microstructural conclusion is drawn from UPV alone.

For treated RTR, UPV changed by an average of −2.6%, −0.4%, and −0.7% in CM-T (28 d), AAM-T (28 d), and AAM-T (56 d), respectively; CM-T at 56 days showed an average increase of 1.0%. No consistent effect of NaOH treatment on UPV was identified across the tested conditions.

### 3.4. Physical Properties of Mortars

The physical properties of the mortar mixes influence the mechanical performance, durability, and weight. These are particularly important when assessing alternative materials like RTR, helping to evaluate their suitability and optimise mix design.

All results on the mortar’s physical properties assessed in this experimental campaign are treated in this section.

#### 3.4.1. Open Porosity and Dry Density

The open-porosity results are presented in Table 8. RCM had open porosity values 11.6% and 14.4% higher than RAAM at 28 and 56 days, respectively. This difference may be influenced by the distinct water contents, curing regimes, and binder systems. Open porosity increased with RTR incorporation; at 20% RTR, values were 11.1–52.3% above the corresponding reference mortar. The increase was larger in CM and CM-T (average +26.9%, +34.5%, and +45.4% for 5%, 10%, and 20% RTR) than in AAM and AAM-T (average +5.6%, +11.5%, and +16.6%). Thus, under the tested conditions, AAM showed a smaller porosity increase after RTR incorporation than CM. No microstructural test was performed to identify the pore-formation mechanism.

Open porosity remained close to the 28-day values at 56 days in CM and AAM. For treated-rubber mixes, the relative changes between treated and untreated counterparts shifted from an average of +0.1% at 28 days to +5.6% at 56 days in CM-T, and from −0.8% to +3.1% in AAM-T. These results are descriptive and do not establish a time-dependent mechanism for NaOH treatment.

At 28 days, treated-rubber mixes had open porosity on average 2.0% lower in CM-T and 2.2% lower in AAM-T than untreated counterparts. At 56 days, the corresponding values were 5.3% and 3.1% higher. The direction of the effect therefore changes with age, and no consistent beneficial effect of NaOH treatment on open porosity was identified.

Dry-density results are presented in Table 9. RCM and RAAM had similar values at 28 and 56 days, with RAAM 2.6% and 2.2% higher, respectively. Dry density decreased with RTR incorporation; at 20% RTR, values were 9.3–18.1% below the corresponding reference mortar. The reductions were larger in CM and CM-T (average −7.9%, −11.0%, and −17.0% for 5%, 10%, and 20% RTR) than in AAM and AAM-T (average −3.0%, −5.6%, and −10.0%). This trend is consistent with replacing denser mineral sand by lower-density rubber and with the measured porosity changes.

Dry density showed only limited age-related variation from 28 to 56 days in all mortars (−0.4% to +1.5%), similar to the small age-related density changes reported by Carvalho et al. [16].

Treated-rubber mixes showed average dry-density changes of −2.1% and −2.8% in CM-T and −0.6% and −0.8% in AAM-T at 28 and 56 days, respectively, relative to untreated counterparts. These differences are small, and no clear influence of NaOH treatment on dry density was identified.

#### 3.4.2. Water Absorption by Immersion

**Figure 9:**
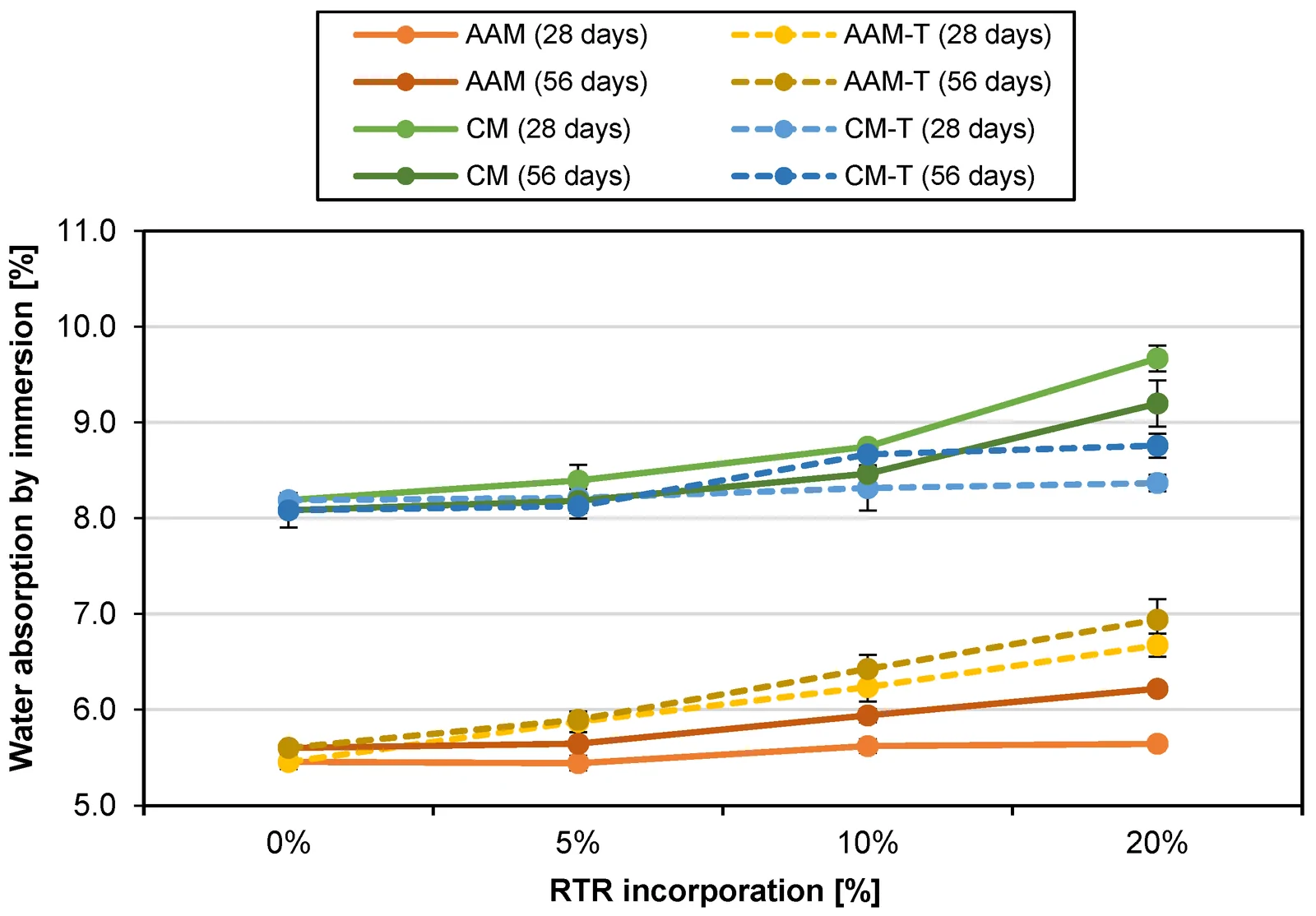
Water absorption (WA) by immersion for mortar mixes.

Water absorption (WA) by immersion results are presented in Figure 9. RAAM had lower values (5.46% and 5.60% at 28 and 56 days) than RCM (8.19% and 8.08%) under the adopted mix-design and curing conditions. WA by immersion generally increased with RTR incorporation; at 20% RTR, values were 2.2–23.9% above the corresponding reference mortar. This trend is consistent with the measured increase in open porosity and with void/compaction effects reported for rubberised materials [7,13]. CM and CM-T values (8.08–9.67%) were higher than AAM and AAM-T values (5.44–6.94%) in the present campaign. These measurements are durability-related indicators of water ingress; broader durability tests were not performed.

From 28 to 56 days, WA by immersion decreased slightly in CM mixes (average −3.0%). In CM-T, AAM, and AAM-T, it increased by an average of +1.6%, +5.6%, and +2.5%, respectively. These are descriptive age-related changes within the tested specimens.

For treated RTR, WA by immersion was on average 6.9% lower at 28 days and 1.0% lower at 56 days in CM-T than in untreated CM mixes. In AAM-T, it was 12.4% and 8.1% higher at 28 and 56 days, respectively. The opposite trends between binder families show that no general effect of NaOH treatment on WA by immersion can be established from this single treatment condition. No chemical or microstructural characterisation was performed to assign these differences to a specific reaction between NaOH and RTR.

#### 3.4.3. Water Absorption by Capillarity

The water absorption by capillarity test was used to calculate the capillary absorption coefficient, with results summarised in Table 10. At 28 days, RCM had a coefficient 13.2% higher than RAAM; at 56 days, RAAM was 11.9% higher than RCM. The coefficient generally decreased with RTR incorporation, except for CM-T at 28 days and AAM-T at 56 days. This trend is consistent with the limited affinity for water generally reported for tyre rubber [10], but it is also affected by pore structure and therefore should not be assigned to rubber hydrophobicity alone.

From 28 to 56 days, the capillary absorption coefficient decreased by an average of 2.8% in CM and 29.0% in CM-T. In AAM and AAM-T, it increased by an average of 85.5% and 123.4%, respectively.

According to the RTR-T with NaOH, there is a tendency for the water absorption coefficient to increase in all mortars when NaOH is used to treat the rubber (average of +149.6%, +25.1%, +36.2%, and +62.6%, respectively, for CM-T (28 d), CM-T (56 d), AAM-T (28 d), and AAM-T (56 d)).

**Figure 10:**
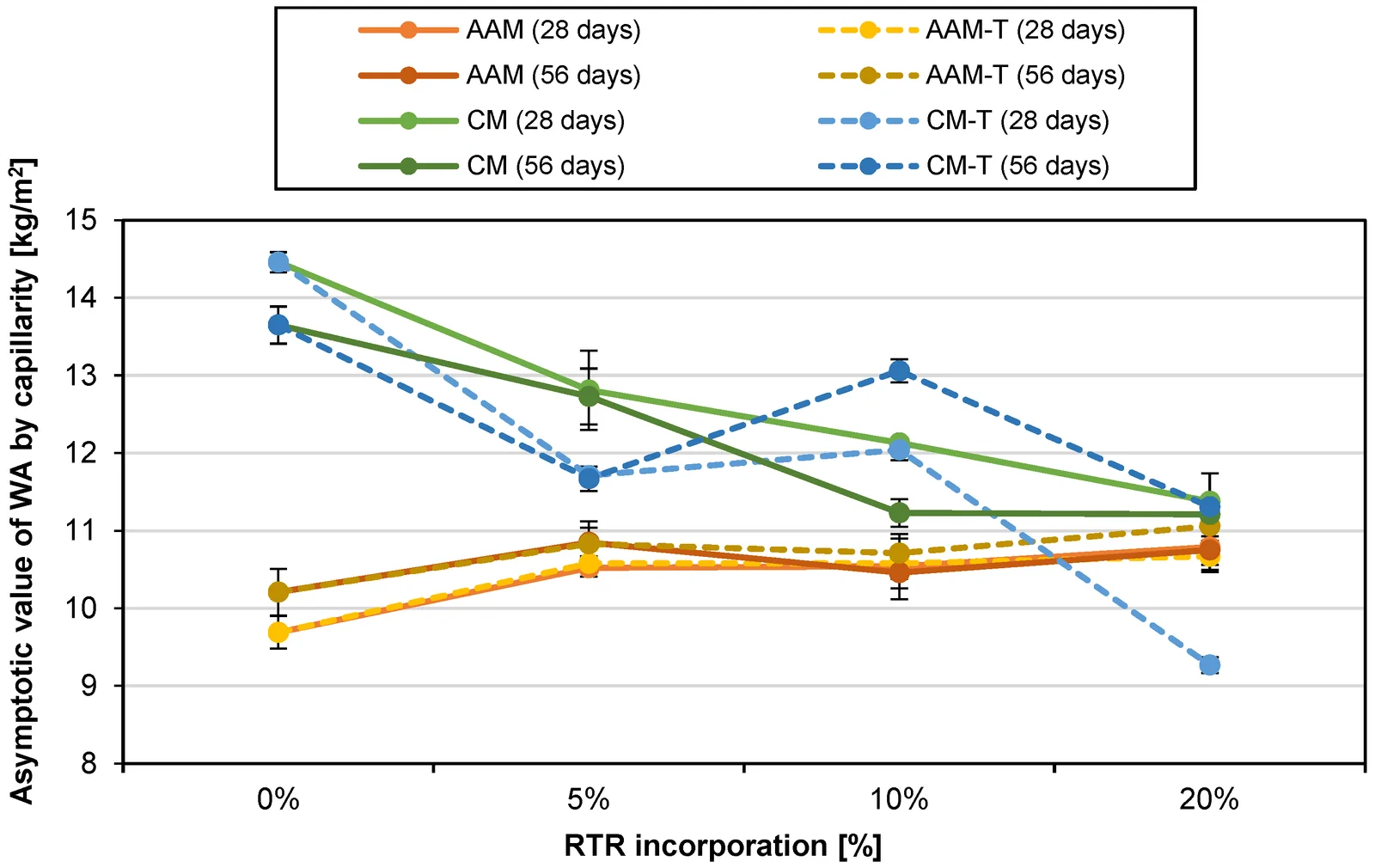
Asymptotic value of water absorption by capillarity for mortar mixes.

The asymptotic values of water absorption by capillarity are summarised in Figure 10. At 28 and 56 days, RCM values were 49.2% and 33.7% higher than RAAM, respectively. This difference may be influenced by the distinct mix designs and measured porosity. In CM and CM-T, the asymptotic value generally decreased with RTR incorporation (average −12.9%, −13.7%, and −23.1% for 5%, 10%, and 20% RTR). Although open porosity increased, the reduced capillary uptake may be consistent with the limited water affinity of rubber reported in the literature [10] and with changes in pore connectivity. In AAM and AAM-T, the asymptotic value increased on average by 7.5%, 6.3%, and 8.8% for 5%, 10%, and 20% RTR, respectively, which may reflect the different pore structure of these mixes.

According to the curing age, the asymptotic value decreases in CM mixes, ranging from 28 to 56 days (average of −3.8%). On the other hand, the asymptotic value increases with the variation in age in CM-T, AAM, and AAM-T (average of +6.1%, +1.8%, and +3.2%, respectively).

According to the RTR-T with NaOH, there is a tendency for the asymptotic value to decrease at 28 days in CM-T with NaOH rubber treatment (average of −9.3%). However, there is a slight increase in the asymptotic value at 56 days in CM-T and AAM-T (average of +3.0% and +1.7%, respectively).

## 4. Conclusions

The main objective of this preliminary experimental research was to evaluate the fresh-state, mechanical, and physical performance of mortars incorporating recycled tyre rubber (RTR) as partial replacement of natural sand (5%, 10%, and 20% by volume), comparing Portland cement mortars (CM) with alkali-activated fly ash mortars (AAM), and assessing one NaOH pre-treatment condition. The conclusions below refer to the adopted mix designs and curing regimes.

- Under the adopted experimental conditions, AAM showed higher compressive and flexural strengths and generally lower open porosity and water absorption than CM. Because the two binder families had different w/b ratios and curing regimes, this difference should be interpreted as a comparison between the tested systems rather than as an isolated binder effect.
- RTR incorporation reduced compressive strength, flexural strength, dynamic modulus of elasticity (DME), and ultrasonic pulse velocity (UPV) as rubber content increased, with the largest losses generally occurring at 20% RTR. These trends are consistent with interfacial-bonding and stiffness-contrast mechanisms reported in previous studies, but the ITZ was not directly characterised here.
- The strength reductions associated with RTR were smaller in AAM than in CM within the tested systems, indicating that the adopted AAM formulation retained a greater proportion of its reference mechanical performance after rubber incorporation.
- The single NaOH pre-treatment condition investigated (saturated solution, 30 min) did not consistently improve mortar performance. Limited strength gains occurred in some 5% RTR mixes, whereas higher rubber contents generally showed lower strengths after treatment. This result applies only to the tested treatment condition and does not demonstrate that NaOH treatment is ineffective in general.
- Fresh and hardened densities decreased as RTR content increased, confirming the expected effect of replacing mineral sand with lower-density rubber. NaOH treatment produced only small and inconsistent density changes.
- Open porosity and water absorption by immersion generally increased with RTR incorporation, particularly in CM. AAM showed lower values for these durability-related indicators in the present campaign; however, drying shrinkage, freeze-thaw resistance, carbonation, chloride penetration, sulphate attack, and wet-dry cycling were not evaluated.
- Capillary-water-absorption results depended on binder family, age, and treatment condition. Although rubber hydrophobicity may contribute to some reductions in capillary uptake, pore connectivity, and other microstructural factors were not measured, and the mechanism cannot be established from the present tests alone.

The results indicate that combining alkali-activated fly ash binders with recycled tyre rubber can support the development of more sustainable mortars, particularly when RTR contents are kept moderate (e.g., 5–10%) to balance weight reduction with acceptable performance. However, the NaOH pre-treatment adopted in this study should not be considered effective under the tested conditions, since it did not consistently improve strength, stiffness, porosity or water absorption. Further research should therefore focus on optimising rubber surface modification strategies, including alternative chemical or mechanical treatments, different NaOH concentrations and treatment durations, and improved washing/drying procedures. Additional microstructural characterisation, such as SEM and MIP, is also recommended to better understand the ITZ and pore structure, together with broader durability assessment involving shrinkage, freeze-thaw resistance, carbonation resistance and chloride-ion penetration resistance. Finally, life-cycle assessment, carbon-footprint calculation, or cost analysis should be performed, as well as the quantification of the environmental burdens associated with NaOH, sodium silicate, and thermal curing.

## Figures and Tables

**Table 1:** Identification of mortar mixes.

Mix ID	Precursor	RTR Incorporation	NaOH Treatment
RCM	Cement	0%	-
RAAM	Fly ash	0%	-
5CM	Cement	5%	No
5AAM	Fly ash	5%	No
5CM-T	Cement	5%	Yes
5AAM-T	Fly ash	5%	Yes
10CM	Cement	10%	No
10AAM	Fly ash	10%	No
10CM-T	Cement	10%	Yes
10AAM-T	Fly ash	10%	Yes
20CM	Cement	20%	No
20AAM	Fly ash	20%	No
20CM-T	Cement	20%	Yes
20AAM-T	Fly ash	20%	Yes

**Table 2:** Composition of cementitious mortar mixes.

Mix ID	RCM	5CM	10CM	20CM
Volume [L]	1	1	1	1
w/b ratio	0.53	0.53	0.53	0.53
Portland cement [g]	430	430	430	430
Fine sand [g]	471.57	448.01	424.41	377.26
Coarse sand [g]	1100.33	1045.50	990.30	880.26
Water [g]	232.15	232.15	232.15	232.15
Superplasticiser [g]	0.86	0.86	0.86	0.86
RTR 0–1 mm [g]	0	21.75	43.45	86.90
RTR 1–2 mm [g]	0	8,10	16.15	32.35
RTR 2–4 mm [g]	0	3.05	6,10	12.25

**Table 3:** Composition of alkali-activated mortar mixes.

Mix ID	RAAM	5AAM	10AAM	20AAM
Volume [L]	1	1	1	1
w/b ratio	0.38	0.38	0.38	0.38
Fly ash [g]	430	430	430	430
Fine sand [g]	458.75	436.01	412.88	367.03
Coarse sand [g]	1070.40	1017.02	963.36	856.32
Water [g]	41.21	41.21	41.21	41.21
Superplasticiser [g]	0.86	0.86	0.86	0.86
NaOH [g]	50.50	50.50	50.50	50.50
Na_2_SiO_3_ [g]	199	199	199	199
RTR 0–1 mm [g]	0	21.15	42.30	84.55
RTR 1–2 mm [g]	0	7.85	15.75	31.45
RTR 2–4 mm [g]	0	2.95	5.95	11.90

**Table 4:** Tests performed in this experimental campaign.

**Aggregates tests**	
**Name**	**Standard**
Particle size distribution analysis	NP EN 933-1 [18], ASTM C33 [19]
Bulk density	NP EN 1097-3 [20]
Real and apparent density	NP EN 1097-6 [21]
Water absorption	NP EN 1097-6 [22]
**Precursors tests**	
**Name**	**Standard**
Bulk density	NP EN 1097-3 [20]
**Fresh state of mortars**	
**Name**	**Standard**
Flow	NP EN 1015-3 [22]
Fresh density	NP EN 1015-6 [23]
**Mechanical-related properties of mortars**	
**Name**	**Standard**
Flexural strength	NP EN 1015-11 [24]
Compressive strength	NP EN 1015-11 [24]
Dynamic modulus of elasticity	ASTM E1876 [25]
Ultrasonic pulse velocity	NP EN 12504-4 [26]
**Physical properties of mortars**	
**Name**	**Standard**
Open porosity	RILEM test No. I.1 [27]
Dry density	RILEM test No. I.2 [28]
Water absorption by immersion	LNEC E-394 [29]
Water absorption by capillarity	NP EN 1015-18 [30]

**Table 5:** Bulk densities of aggregates and precursors.

Material	Bulk Density Measurements [kg/m^3^]	
	Average	Stand. Deviation
Fine sand (0–2 mm)	1493.3	±6.3
Coarse sand (0–4 mm)	1514.5	±2.4
Recycled tyre rubber	433.6	±7.3
Portland cement	1039.1	±4.7
Fly ash	981.0	±1.8

**Table 6:** Densities and water absorption of aggregates.

Material	Real Density ρ_rd_ [kg/m^3^]	Apparent Density ρ_a_ [kg/m^3^]	Saturated Surface Density ρ_ssd_ [kg/m^3^]	WA 24 h [%]
Fine sand (0–2 mm)	2574.8	2594.9	2582.5	0.3
Coarse sand (0–4 mm)	2565.6	2610.5	2582.7	0.7
Recycled tyre rubber	987.8	1025.5	1024.5	3.7

**Table 7:** Fresh density for mortar mixes.

Mix	Fresh Density Results [kg/m^3^]			
	0%	5%	10%	20%
CM	2188.0 ± 22.1	2030.6 ± 17.6	1952.6 ± 41.2	1817.3 ± 13.2
CM-T	2188.0 ± 22.1	1989.8 ± 19.1	1885.0 ± 15.7	1771.2 ± 20.1
AAM	2219.7 ± 33.5	2217.0 ± 23.4	2172.7 ± 32.0	2080.3 ± 23.6
AAM-T	2219.7 ± 33.5	2197.7 ± 25.6	2172.0 ± 27.6	2092.2 ± 14.5

**Table 8:** Open porosity results for mortar mixes.

Mix	Open Porosity Results [%]			
	0%	5%	10%	20%
CM (28 d)	19.3 ± 0.1	25.3 ± 0.2	26.9 ± 0.7	29.4 ± 0.2
CM-T (28 d)	19.3 ± 0.2	25.0 ± 0.0	26.5 ± 0.4	28.4 ± 0.2
AAM (28 d)	17.3 ± 0.1	18.9 ± 0.4	20.3 ± 0.2	20.5 ± 0.1
AAM-T (28 d)	17.3 ± 0.2	18.3 ± 0.1	19.3 ± 0.0	20.8 ± 0.1
CM (56 d)	20.7 ± 0.1	25.0 ± 0.6	26.1 ± 0.4	28.6 ± 0.9
CM-T (56 d)	20.7 ± 0.3	26.1 ± 0.2	28.0 ± 0.2	29.8 ± 0.1
AAM (56 d)	18.1 ± 0.1	18.9 ± 0.3	19.1 ± 0.3	20.1 ± 0.4
AAM-T (56 d)	18.1 ± 0.3	18.6 ± 0.2	20.2 ± 0.0	21.1 ± 0.3

**Table 9:** Dry density results for mortar mixes.

Mix	Dry Density Results [kg/m^3^]			
	0%	5%	10%	20%
CM (28 d)	2048.0 ± 3.9	1909.3 ± 4.4	1843.3 ± 18.1	1718.6 ± 4.6
CM-T (28 d)	2048.0 ± 5.5	1873.4 ± 6.9	1803.4 ± 8.9	1681.4 ± 3.5
AAM (28 d)	2101.8 ± 3.9	2043.0 ± 15.3	1971.6 ± 4.5	1899.1 ± 3.7
AAM-T (28 d)	2101.8 ± 5.5	2032.1 ± 2.4	1974.9 ± 3.5	1869.7 ± 3.2
CM (56 d)	2053.5 ± 6.7	1909.4 ± 7.3	1856.9 ± 13.3	1728.8 ± 18.3
CM-T (56 d)	2053.5 ± 3.3	1866.8 ± 4.2	1796.5 ± 2.3	1681.1 ± 7.6
AAM (56 d)	2098.9 ± 6.7	2039.4 ± 8.4	2000.7 ± 7.4	1904.5 ± 10.1
AAM-T (56 d)	2098.9 ± 3.3	2033.4 ± 2.1	1981.1 ± 12.9	1885.5 ± 9.8

**Table 10:** Coefficient of capillarity results for mortar mixes.

Mix	Coefficient of Capillarity Results [kg/(m^2^ s^1/2^)]			
	0%	5%	10%	20%
CM (28 d)	0.043 ± 0.002	0.028 ± 0.000	0.020 ± 0.007	0.019 ± 0.001
CM-T (28 d)	0.043 ± 0.001	0.055 ± 0.001	0.061 ± 0.002	0.047 ± 0.000
AAM (28 d)	0.038 ± 0.002	0.026 ± 0.004	0.019 ± 0.003	0.017 ± 0.004
AAM-T (28 d)	0.038 ± 0.001	0.030 ± 0.003	0.027 ± 0.003	0.020 ± 0.005
CM (56 d)	0.059 ± 0.003	0.025 ± 0.002	0.018 ± 0.010	0.018 ± 0.002
CM-T (56 d)	0.066 ± 0.002	0.049 ± 0.000	0.042 ± 0.002	0.027 ± 0.011
AAM (56 d)	0.059 ± 0.003	0.023 ± 0.001	0.033 ± 0.001	0.024 ± 0.003
AAM-T (56 d)	0.066 ± 0.002	0.057 ± 0.001	0.058 ± 0.003	0.063 ± 0.002

## Data Availability

The original contributions presented in this study are included in the article. Further inquiries can be directed to the corresponding author.

## Abbreviations

The following abbreviations are used in this manuscript:

AAM	Alkali-activated mortar
AAM-T	Alkali-activated mortar with NaOH-treated rubber
CM	Portland cement mortar
CM-T	Portland cement mortar with NaOH-treated rubber
RAAM	Alkali-activated reference mortar
RCM	Portland cement reference mortar
RTR	Recycled tyre rubber
RTR-T	Recycled tyre rubber treatment
DME	Dynamic modulus of elasticity
UPV	Ultrasonic pulse velocity
WA	Water absorption
